# Supplementary material for: Content-rich biological network constructed by mining PubMed abstracts
Source: BMC Bioinformatics. 2004 Oct 8;5:147. doi: 10.1186/1471-2105-5-147 (PMC528731; doi:10.1186/1471-2105-5-147)
Supplement: Additional File 5 — The original Chilibot query results of the term "long-term potentiation (LTP)" and 22 other terms, limiting the latest references analyzed to the years 1990, 1995, 2000, and 2004. [file 1471-2105-5-147-S5.bz2 › chilibotAdditionalFile5/ltp2000/html/TRKB.html]

 


**TRKB** (Input: TRKB ) 

---


**Google Searches:** Entire Web  | EDU domain only  | PDF files only

.

**External Links:** OMIM |
LocusLink
| Swissprot
| GeneCards

  
**Maps of TRKB**

Simple
Complete
graph in
radiant
tree
square
layout.

**New Hypothesis !**

**Synonyms** 

- trk b   [PubMed]

- trkb   [PubMed]

**Synopsis**

- These results suggest that two different pathways, the c ras and the PLC gamma pathway, are activated by **TrkB** receptors in primary neurons.  J Neurochem, 1995 
  [23]

- Finally, the presence of mRNA transcripts for the different neurotrophins and neurotrophin receptors, with the exception of Trk B  [**TRKB**] , is shown by reverse transcription polymerase chain reaction RT PCR on RNA extracted from freshly isolated rat hepatic stellate cells, compared with hepatocyte RNA.  Hepatology, 2001 
  [20]

- **TrkB** IgG or K252a itself completely blocked LTP, suggesting that endogenous BDNF or another **TrkB** ligand plays a role in LTP in the developing visual cortex.  J Neurosci, 1997 
  [19]

- Activation of **trkB** modulates hippocampal long term potentiation  [LTP] , in which postsynaptic N methyl D aspartate glutamate receptors play a key role.  Proc Natl Acad Sci U S A, 1997 
  [19]

- Thus, both BDNF and its receptor **trkB** are induced in granule cells by stimulation that produces durable LTP, suggesting that this neurotrophin and its receptor play an important role in memory formation and may be suitable targets for the development of cognitive enhancing drugs in the treatment of diseases, such as Alzheimer s.  Brain Res Mol Brain Res, 1997 
  [16]

- Firstly, we show that while BDNF and **trkB** are induced as immediate early genes following injury, the injury induced expression of activinA and trkC may be regulated by ITFs.  Prog Neurobiol, 1999 
  [16]

- Although the expression of BDNF and its receptor **TrkB** still occurs in the adult stage, their physiological role in the mature CNS is NOT fully understood.  J Neurosci, 1997 
  [16]

- Taken together, these results indicate that endogenous Trk B  [**TRKB**]  ligands are necessary for the expression of LTP  Eur J Neurosci, 2000 
  [14]

- These results indicate that the **TrkB** Shc site mediates both neuronal survival and axonal outgrowth by activating the P13 kinase and MEK signaling pathways.  Neuron, 2000 
  [14]

- These results indicate a causal link of synapsin phosphorylation via BDNF, **TrkB** receptors and MAP kinase with downstream facilitation of neurotransmitter release.  Nat Neurosci, 2000 
  [14]

- However, both **TrkB** IgG fusion proteins and BDNF antibody similarly reduced LTP, suggesting that only BDNF but no other ligands of the **TrkB** receptor are likely to be involved in LTP induction.  J Neurosci, 1999 
  [13]

- We found that homo and heterozygous mutant mice show no difference in the induction rate or magnitude of E LTP and L LTP induced by theta burst or tetanus stimulation, suggesting that the Shc binding site in the **TrkB** receptor and its downstream activated signaling cascade is NOT involved in hippocampal synaptic plasticity.  Neuropharmacology, 2000 
  [13]

- Specifically, **trkB** and NT 3 mRNA elevations were restricted to granule cells in the ipsilateral dentate gyrus.  J Comp Neurol, 1996 
  [13]

- The PSD localization of both BDNF and **trkB** supports the contention that this receptor ligand pair participates in postsynaptic plasticity.  J Neurosci Res, 2000 
  [10]

- We have generated conditionally gene targeted mice in which the knockout of the **trkB** gene is restricted to the forebrain and occurs only during postnatal development.  Neuron, 1999 
  [10]
